# Comparison of Food Compound Intake Between Food-Allergic Individuals and the General Population

**DOI:** 10.3390/nu17142297

**Published:** 2025-07-11

**Authors:** Meike E. Vos, Marie Y. Meima, Sabina Bijlsma, W. Marty Blom, Thuy-My Le, André C. Knulst, Geert F. Houben

**Affiliations:** 1 The Netherlands Organization for Applied Scientific Research (TNO), Princetonlaan 6, 3584 CB Utrecht, The Netherlandsmarty.blom@tno.nl (W.M.B.); geert.houben@tno.nl (G.F.H.); 2 Department of Gastroenterology and Hepatology, University Medical Center Utrecht, 3584 CX Utrecht, The Netherlands; 3 Center for Translational Immunology, University Medical Center Utrecht, 3584 CX Utrecht, The Netherlands; sabina.bijlsma@tno.nl (S.B.);; 4 Department of Dermatology and Allergology, University Medical Center Utrecht, Utrecht University, 3584 CX Utrecht, The Netherlands

**Keywords:** food allergy, food compounds, food composition databases, NEVO, FooDB, cohort data

## Abstract

**Background:** Individuals with food allergies typically need to avoid specific allergens, leading to distinct dietary choices. Their food product intake may therefore vary from that of the general population, potentially leading to differences in their intake of nutrients and other food compounds. **Methods:** We compared food compound and nutrient group intakes between the general Dutch adult population (*n* = 415) and food allergic Dutch adult patients with either milk and/or egg allergies (*n* = 16), peanut and/or tree nut allergies (*n* = 35) or a combination of milk/egg and peanut/tree nut allergies (*n* = 22). We translated 24-hour dietary recall data into food compound intake values. We used a mixed effects ANOVA model and considered compound intakes statistically significantly different at FDR-corrected *p* < 0.05. Additionally, compounds with uncorrected *p* < 0.01 were explored for potential relevance. **Results:** A total of 489 compounds or nutrient groups were included in the statistical analysis. Milk/egg and mixed allergic patients had significantly lower intakes of beta-lactose, butyric acid, caproic acid, caprylic acid, capric acid, lauric acid, myristic acid, myristoleic acid, conjugated linoleic acid, and remainder saturated fatty acids (*p* < 0.05, FDR corrected), with mean intake factors of 1.6–3.2 and 1.3–2.9 lower, respectively, than the general population. In addition, 36 other compounds showed intake differences with a *p* < 0.01 without FDR correction. There were no statistically significant differences between the peanut/tree nut allergy group and the general population. **Conclusions:** Our study shows significantly lower intakes of 10 mainly dairy-derived compounds by the milk/egg and mixed-allergic patients, presenting the potential for long-term health consequences and the need for supplementation a relevant consideration, warranting further research.

## 1. Introduction

Many individuals suffer from food allergies, limiting them in their food choices and warranting constant vigilance toward seemingly harmless products. Food allergies are associated with symptoms such as acute swelling of the lips and throat, nausea, vomiting, dyspnea and a drop in blood pressure upon ingesting the allergen [1,2]. To avoid these symptoms, patients have to adhere to a diet that is completely void of the products containing the culprit allergens. Adherence to a diet can be challenging, as certain allergens are common ingredients in many products to date. Also, many products may contain traces of allergens, forcing food-allergic individuals to avoid these [3,4]

Avoiding products that contain allergens may limit food allergic individuals’ intake of essential nutrients and other dietary factors such as fatty acids, polyphenols and fibers [5]. Cohort studies in children have shown that nutrient deficiencies pose a significant health risk [5,6,7,8]. The potential undernutrition of the adult allergic population has not been studied as widely as that of children. In adults, undernutrition may not affect development as much as in children, but low intakes of vital nutrients can still lead to health issues such as anemia, osteoporosis, and cardiovascular disease [6].

Nutrient intake may differ between food allergic and general populations, but there are also many compounds in food that influence health beyond the well-studied essential nutrients. While the effects of avoidance of these may be more subtle, they may contribute to long-term health outcomes. For example, phenolic compounds are well-researched for their anti-inflammatory effects and are suspected to have various biological and synergistic roles. Some have also been linked to improved cardiovascular health and anti-diabetic properties [9,10]. It is suspected that the human diet consists of tens of thousands of different chemicals, many of which have not or have hardly been studied in the context of consumption or health. The fact that these compounds are not considered in food intake and health research is a major gap in current research. Including more compounds in studies could contribute to a more comprehensive view of differences in food compound intake between populations and their potential health effects [11,12].

This study provides a comparison of food intake between Dutch adult food allergic (FA) patients and the general Dutch population, both on nutrient group and individual food-compound level.

## 2. Methods

### 2.1. Cohort Data

Food intake data for the FA groups were collected in the Netherlands by the University Medical Center Utrecht (UMCU) and the Netherlands Organization for Applied Scientific Research TNO during 2016 and 2017. Patients were physician-confirmed diagnosed with a hen’s egg, cow’s milk, peanut or tree nut allergy based on the patient’s convincing history of allergic complaints to the food and a positive skin prick test and/or serum specific IgE and/or a positive food challenge [13]. The patients were assigned into three groups: a group with a cow’s milk and/or hen’s egg allergy (*n* = 16), a group with a peanut and/or tree nut allergy (*n* = 35), and a group with both cow’s milk and/or hen’s egg AND peanut and/or tree nut allergies, the latter hereafter referred to as the mixed allergy group (*n* = 22). Food intake data for the general population was obtained from the Dutch National Institute for Public Health and the Environment (RIVM). These data are collected in a four-yearly recurring study called the Voedsel Consumptie Peiling (VCP). The VCP for 2012–2016 [14] was used in this study to maintain coherence with food intake data of the FA groups. The FA patients and subjects from the general population were interviewed by experts from the UMCU or the RIVM, respectively, to assess their nutritional intake using a structured 24 h dietary recall for two non-consecutive days, one on a weekday and one on a weekend day [13]. Participants were ≥19 years of age and supplements or medicine use were not included in the analysis in either population.

Participants in the general population were selected based on age, education level, and sex to match the proportions of the FA groups. In addition, individuals from the general population who reported following a diet because of an allergy were excluded. Ultimately, the general population sample was reduced from 2078 to 415 participants based on these criteria. For certain purposes within this research, we used the complete population of 2078 participants, i.e., for compound selection (see Section 2.4) and homogeneity control (Section 2.5). The general characteristics of the study populations are shown in Table 1.

### 2.2. Food Compound Databases

Dietary data were first translated to intake levels of food compounds and nutrient groups. To this end, two food composition databases were employed: the Dutch food composition database, called Nederlands Voedingsstoffenbestand (NEVO), and the international food composition database FooDB.

NEVO includes plant- and animal-based food items, beverages and processed items. The database contains concentration levels for 100 individual compounds for each food item, covering micro- and macronutrients and a selection of fatty acids. It also provides concentration levels for 37 nutrient groups, including carbohydrates, fatty acids, proteins and fibers (e.g., “saturated fatty acids, total”). We used the latest NEVO update from 2023 in our study [15]. As the participant data were previously collected with NEVO version 2016 [14], not all reported NEVO food items were present in the version of 2023. The missing food items from the version of 2016 were therefore manually added to the version of 2023.

Food compound data of FooDB, last updated on 7 April 2020, was downloaded from www.foodb.ca, on 13 December 2023 [16]. FooDB is a freely available, aggregated database containing data from 371 different sources, most of which are from scientific literature. The downloaded data contained information on 10,898 compounds and nutrient groups for 9461 food items, including amino acids and secondary plant metabolites, which are not included in NEVO. Not all data were complete: concentration levels, compound names, or food item names were occasionally missing and 775 food items and 8332 compounds were therefore removed. Additionally, FooDB contained compound data for inedible plant parts, different nomenclature for identical compounds and duplicated information. FooDB was thoroughly curated prior to analysis.

### 2.3. Coupling Food Items from FooDB to NEVO

Since the food intake data from VCP and our FA patients were NEVO-coded, NEVO served as the starting point. To substantiate compound data for the foods consumed by participants, corresponding FooDB items were matched to the NEVO-coded food items. Coupling of the items was carried out using word embeddings, retrieved from OpenAI in March 2024 (OpenAI Platform) [17]. Word embeddings are mathematical representations of the semantics of a word, ensuring that unsimilar words with similar meanings, could be matched automatically (e.g., “eggplant” and “aubergine”). Word-embedding resemblance was assessed by calculating cosine similarity between the embeddings [18]. The limit of possible matches retrieved by this method was arbitrarily set at 150, thus yielding 150 best resembling matches for each NEVO item. From this list, final matches were selected manually using the strategy described in Meima et al. (2023) [12]. Briefly, matches were labelled according to their level of similarity, taking into account processing differences between matched items. One deviation from the matching strategy from Meima et al. (2023) is that we did not set a limit for the number of food item matches per NEVO item [12].

### 2.4. Calculation of the Average Daily Intakes

For reported NEVO food items that were matched to a FooDB item, compound and nutrient data were available from both databases. Compound concentration values from NEVO and FooDB were averaged per item or nutrient group when multiple values were available. If, for a NEVO item, a suitable FooDB match was not present, compound and nutrient data from NEVO was available for this item. This process yielded a final concentration for each compound and nutrient group for each food item, which was then used to calculate the participants’ intake values.

Each participant reported their consumption of food items in grams. This amount was multiplied by the compound or nutrient group concentrations (in mg/g) for the respective food item. The compound intake values derived from consumption of different food items and different eating occasions per day were summed for each reporting day, and the average of the two reporting days was calculated, resulting in the average daily compound intake in mg.

### 2.5. Removal and Handling of Skewed Data

**Figure A1:**
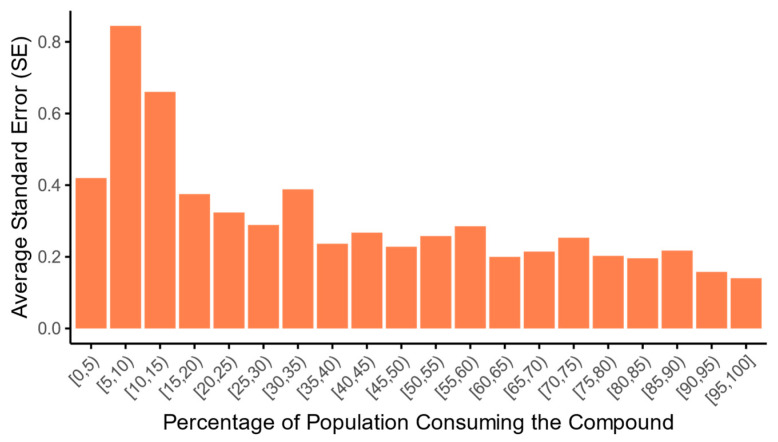
The average standard error of the mean intake difference between the allergic population and the general population, per level of consumption of compounds. A higher proportion of consumers means higher reliability.

**Figure A2:**
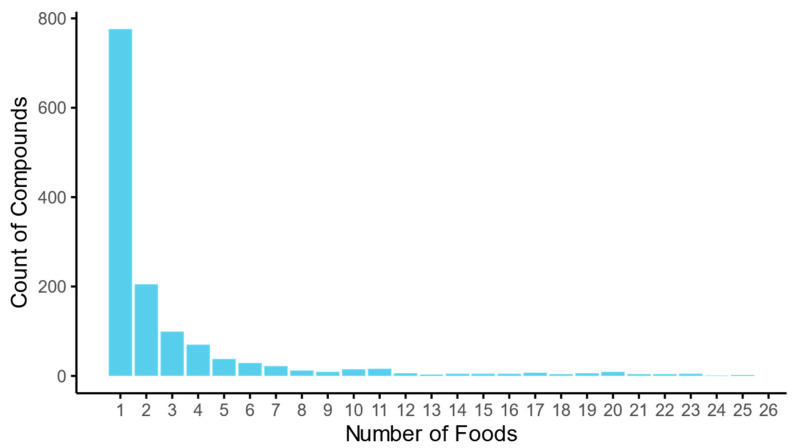
Distribution of food compounds per number of foods in which they occur.

To ensure reliable results, only compounds consumed by a sufficient number of participants were selected for statistical analyses. We set a criterion for each compound that it should be consumed by at least 40% of the population to be included in the final analyses to ensure reliable estimates, based on obtaining relatively low and stable standard errors (Figure A1, Appendix A). This criterion led to a reduction in compounds and nutrient groups from 1626 to 489, as many compounds were present in only a few food items and therefore not regularly consumed (Figure A2, Appendix A).

Some beverages caused skewed compound intake distributions, particularly for polyphenols found in coffee, tea and beer. Upon reviewing the food consumption data, we found that certain study participants had consumed substantially more of these beverages than others. These outliers in beverage intake persisted on a logarithmic scale and were therefore removed from the dataset. To retain as much information as possible, only the beverage data was removed and not the entire participant. This was performed for individuals who had mentioned consuming the beverage more than three times the standard deviation above the mean. For coffee (mean: 4.9 consumptions over 2 days; SD: 3.9), this was the case for 17 individuals, for tea (mean: 3.1; SD: 3.7) for 32 individuals, and for beer (mean: 0.6; SD: 1.6) for 54 individuals. The selection was carried out using the non-adjusted general population plus FA groups (*n* = 2151).

### 2.6. Statistical Analysis

Prior to adjustment for age, sex, and education of the general population to align with the characteristics of the allergic population, homogeneity scatterplots of model residuals were created based on a linear model prediction for each of the four groups. Over a hundred scatterplots were manually scrutinized to ensure homogenic results. A logarithmic scale was applied to minimize outliers and force homogeneity of model residuals as much as possible.

ANOVA was used to identify statistically significant differences in compound and nutrient group intake among the groups. Subsequently, in case of statistically significant differences, post hoc tests were performed to determine which specific populations exhibited differences in their intakes. Because the allergic groups were rather small (milk/egg, *n* = 16; peanut/tree nut, *n* = 35; mixed, *n* = 22), and the general population was more than ten times larger (*n* = 415), a Monte Carlo simulation was applied to account for sample size. The ANOVA and post hoc tests were carried out a thousand times, each time with a different sub-population from the general population. This sub-population consisted of 73 subjects, reflecting the total sample size of the allergic groups. From the 1000 iterations, average *p*-values and effect sizes were calculated. Differences were considered statistically significant if *p* < 0.05 after FDR correction. Additionally, to capture potentially relevant differences, we explored compounds with *p* < 0.01 without FDR correction. All analyses were performed in R version 4.4.2 using the emmeans package for building the statistical model and conducting the post hoc tests.

We used ChatGPT (GPT-3.5) to assist with language editing during the preparation of this manuscript. All content generated with the tool was reviewed and revised by the authors to ensure accuracy and clarity.

## 3. Results

### 3.1. Food Item Matches

A total of 1768 food items reported by participants were matched to items from FooDB. For 628 items, no similar items in FooDB were found and therefore could not be matched. Yet, for these items, NEVO information was still available. The number of FooDB matches per NEVO item was mostly three (e.g., the target item “brazil nuts unsalted” was matched to the FooDB items “brazil nut”, “brazil nuts” and “brazilnut”), with a median of 5. A total of 489 compounds complied to the criterium of being consumed by at least 40% of the population and could be included in the final analysis.

### 3.2. Statistical Comparison of Food Compound and Nutrient Group Intakes with FDR Correction

The Monte Carlo ANOVA resulted in nine compounds and one nutrient group for which at least one statistically significant difference was found between any of the groups under FDR correction (*p* < 0.05). Both the milk/egg and mixed allergy groups had a significantly lower intake of beta-lactose, butyric acid, caproic acid, capric acid, caprylic acid, lauric acid, myristic acid, myristoleic acid, conjugated linoleic acid and the nutrient group remainder saturated fatty acids (i.e., the part of saturated fatty acids that were not specified) compared to the general population. The mean intakes of these compounds were factors of 1.6–3.2 lower in the milk/egg allergic group and factors of 1.3–2.9 lower in the mixed allergy group, compared to the general population. No statistically significant differences were found between the peanut/tree nut allergy group and the general population (*p* ≥ 0.05, FDR corrected). Table 2 presents the intake values in mg/day (mean and standard deviation), ANOVA, and post hoc test results, for the compounds with statistically significant differences.

### 3.3. Statistical Comparison of Food Compound and Nutrient Group Intakes Without FDR Correction

To provide a comprehensive view of the data, we also examined compounds that were not significant after FDR correction but showed a non-corrected *p*-value below 0.01 (Table A1, Appendix B). In addition to the statistically significant differences found with *p* < 0.05 after FDR correction, the mixed allergy group had a significantly lower intake of calcium, cholesterol, and animal protein, with all factors of 1.4 lower intakes compared to the general population. The mixed allergy group had 1.7- to 1.8-fold higher intakes of 24 compounds found in bell pepper compared to the general population, most of which were capsianosides, capsaicinoids, and their derivatives. Furthermore, we observed statistically significant higher intakes for the milk/egg allergy group for the nutrient group dietary fiber (with 1.3-fold higher intake compared to the general population) and two soy polyphenols, i.e., daidzein and genistein (with higher intake levels of 4.6- and 4.7-fold than in the general population, respectively). Again, no statistically significant differences were found between the peanut/tree nut allergy group and the general population.

## 4. Discussion

We analyzed the intake of food compounds and nutrient groups among three food allergy (FA) groups and compared these to the general population. We showed that individuals with milk or egg allergies, as well as those with milk or egg AND peanut or tree nut allergies, have significantly different intakes of several compounds compared to the general population. Our findings reflect dietary patterns within a Western diet context, where (high-fat) dairy products are commonly consumed.

The FDR-corrected results all indicated a statistically significantly lower intake by the milk/egg and mixed allergy group for the compounds beta-lactose, seven short/medium-chain fatty acids (SCFA/MCFA), conjugated linoleic acid and the nutrient group saturated fatty acids remainder. All these components are highly prevalent in dairy products, especially in fatty cheese, and these findings are therefore in line with the dietary restrictions (i.e., avoidance of dairy products) of the individuals in these allergy groups [8,19,20].

To the best of our knowledge, the large difference in intakes of SCFA and MCFA by allergic individuals in comparison to that by the general population has not been described previously. The intakes by the allergic groups were up to a factor of 3 lower than those of the general population. E.g., the general population consumed on average 652 mg of butyric acid, whereas the milk/egg allergic group consumed only 266 mg on average. This low intake of SCFA/MCFA may have health consequences in allergic individuals. SCFAs are known to reduce intestinal inflammatory activity [21], and MCFAs contribute to energy supply in insulin-resistant tissues, potentially enhancing brain metabolism and playing a role in Alzheimer’s disease prevention [22]. Additionally, they may improve glucose metabolism, offering potential benefits for obesity management [23]. In part, SCFAs and MCFAs are produced by gut bacteria when digesting fiber, of which the milk/egg allergic group had a significantly higher intake than the general population (non-FDR-corrected *p* < 0.01). High fiber intake could therefore possibly have partly compensated for low SCFA/MCFA intake in the milk/egg allergy group.

The results without FDR correction and *p* < 0.01 showed a similar trend as the FDR-corrected results described above, yet with additional compounds differing in intake between the allergic and non-allergic populations. The mixed allergy group had a factor 1.5 lower intakes of compounds that can also be linked to low intake of dairy products and eggs, including calcium, animal protein, and cholesterol. The milk/egg allergy group had considerable higher intakes, 4.6–4.7-fold higher respectively, of the soy polyphenols daidzein and genistein, which is likely due to a higher intake of dairy replacement options sourced from soy. The milk/egg allergy group also had a 1.3-fold higher intake of fiber, as mentioned, and the mixed allergy group had 1.7–1.8-fold higher intake of twenty-four compounds found only in bell pepper.

It is known that due to their reduced intake of dairy products, milk-allergic patients are often advised to take supplementary calcium to ensure they meet their nutritional needs. It should be noted that supplementation was not taken into account in the dietary questionnaire used in our study. It is therefore not known whether, but plausible that, (a part of) the milk allergic patients in our study compensated the lack of calcium intake through food items by supplementation.

The lower intake of cholesterol by the mixed allergy group compared to the general population was likely due to their reduced or absent consumption of eggs. Generally speaking, reducing dietary cholesterol can help lower levels of low-density lipoprotein cholesterol, which is beneficial for cardiovascular health [24].

The higher intake of the soy polyphenols daidzein and genistein by the milk/egg allergy group was due to higher intake of soy-based dairy alternatives. To the best of our knowledge, our study is the first to highlight the increased presence of these polyphenols in the diets of individuals with milk or egg allergies. Previous studies suggest that long-term higher intake of genistein and daidzein could help prevent obesity and reduce cardiovascular risk over time [25,26,27]. Additionally, we assume that the higher intake of soy products by milk allergic individuals may explain why no significant difference in calcium intake was found for the milk/egg allergy group, as soy products are often fortified with calcium [28,29].

The higher intake of twenty-four compounds unique to bell peppers by the mixed allergy group compared to the general population were primarily capsianosides and capsaicinoids. Indeed, 64% of the participants in the mixed allergy group had consumed at least one serving of bell pepper within the two days they were interviewed, compared to only 34% in the general population. A plausible hypothesis for this difference in bell pepper consumption is that individuals in the mixed allergy group, due to their diverse allergies, tend to consume more unprocessed foods such as vegetables, as prepackaged foods have a higher likelihood of containing (undisclosed) allergens. This hypothesis is supported by the higher (yet not significant) mean intake of vitamins and minerals found in vegetables in the mixed allergy group.

No statistically significant differences were observed between the general population and the peanut/tree nut allergy group. It is likely that the lack of significant differences is due to a relatively lower frequency of peanut and tree nut as main ingredients in the diet. Milk and eggs are more frequently consumed in the general population and are also more common ingredients in many processed foods [30]. Consequently, individuals with a peanut/tree nut allergy likely can consume a wider variety of (processed) foods compared to those with milk/egg allergies. As a result, the peanut/tree nut allergy group may have a dietary pattern more similar to the general population. This similarity might also explain why the intake of minerals and vitamins from vegetables is comparable to that of the general population, and consistently lower, though not significantly, than those of the mixed and the milk/egg allergy groups.

A strength of this study is that the food intake data was collected by means of 24 h dietary recalls, a highly accurate method validated and conducted by trained dieticians. In addition, 489 compounds were analyzed, which is substantially more than in similar studies. For instance, D’auria et al. (2022) and Maslin et al. (2018) studied the nutrient intake of food allergic populations (children and adults, respectively) using food diaries, and included not more than 25 compounds [8,31]. Our extensive analysis was made possible by using FooDB, following a thorough data-cleaning process, and applying artificial intelligence to match NEVO items with FooDB items.

This research has its limitations. The patients in our study were divided into three groups based on their allergy profiles. However, these were not the only allergies present; some participants across the three groups might have suffered from other allergies as well, such as soy, sesame, or fruit allergies. These differences could not be accounted for in this study as it was partly unknown which participant had which additional allergies. Also, further splitting up the groups according to additional allergy profiles would result in sample sizes too small to conduct the type of analyses performed in this study.

The relatively small size of the allergy groups may have influenced the results. All FDR-corrected significant differences present in the milk/egg allergic group were also present in the mixed population. However, animal protein and cholesterol showed non-FDR-corrected significance only in the mixed allergy group, which would be expected in the milk/egg allergy group as well. This may have been due to the smaller sample size of the milk/egg allergy group. To address the small food-allergic groups, we adjusted for age, sex, and education proportions based on the general population and applied a Monte Carlo simulation. While the adjustments and simulations were effective, the small size of the milk/egg allergic group may still have influenced the results. Despite observing large differences when comparing means, the limited number of participants might have prevented these differences from being statistically significant.

Supplement use was not assessed in this study, which may limit the comprehensiveness of total nutrient-intake estimates. However, since the primary goal of the study was not to examine associations with health outcomes, this limitation is less critical and allows for a clearer view of nutrient intake from food sources alone.

Lastly, although our study included a large number of compounds, it still represents only a limited fraction of the total of the estimated tens of thousands of compounds present in food, of which a large part has not been identified yet or has not been properly stored in food composition databases [12]. While we discussed the potential health implications of several compounds above, a complete understanding of the overall net health effect would require consideration of many more or all food compounds present in the diet.

## 5. Conclusions

In this study, we applied a new method to compare food compound intakes between allergic individuals and the general population, aiming at identifying possible nutritional deficiencies. Notably, milk/egg and mixed allergic individuals showed a significantly lower intake of SCFA and MCFA that are presumed to play a beneficial role in the human body, including intestinal health. The low intake of these fatty acids may therefore potentially negatively affect their health. On the other hand, lower cholesterol and higher total fiber, genistein and daidzein intakes in the mixed allergic group might provide a health benefit over the general population. Further research is needed to better understand the long-term health implications of these intake differences for allergic individuals, which could ultimately guide dietitians in making more informed dietary recommendations for individuals that suffer from food allergies.

## Figures and Tables

**Table 1:** General characteristics of food allergic patients and the general population.

		Allergy Groups *n* (%)	General Population *n* (%)
Total		73	415
Allergies	Cow’s milk or hen’s egg	16 (22)	
	Peanut or tree nut	35 (48)	
	Mixed	22 (30)	
Sex	Female	51 (70)	265 (64)
	Male	22 (30)	150 (36)
Age	19–30	21 (29)	144 (34)
	31–50	37 (51)	202 (49)
	51–69	15 (20)	69 (17)
Education	Low	4 (5)	23 (5)
	Middle	20 (27)	117 (28)
	High	47 (64)	275 (66)

**Table 2:** Intake levels of compounds (mg/day) for which the allergic population showed statistically significant different intakes compared to the general population.

			General Population	Allergy Groups		
Compound	*p*-Value ANOVA, FDR Corrected		No Allergy ^a^ (*n* = 415)	Cow’s Milk or Hen’s Egg (*n* = 16)	Peanut or Tree Nut (*n* = 35)	Mixed (*n* = 22)
Beta-Lactose	<0.0001	Mean	10,463	3448	7553	3860
		SD	10,031	6221	6035	8830
		Post hoc *p*-value		<0.0001 *	0.558	<0.0001 *
Fatty Acid 10:0, Capric Acid	<0.0001	Mean	663.2	375.9	714.1	415.6
		SD	428.8	496.3	715.0	504.0
		Post hoc *p*-value		<0.0001 *	0.392	<0.00010 *
C18:2 cis trans	0.001	Mean	33.40	10.45	29.56	11.64
		SD	35.47	18.38	30.71	23.12
		Post hoc *p*-value		0.005 *	0.680	<0.0001 *
Fatty Acid 12:0, Lauric Acid	0.016	Mean	1272	595.1	1106	963.5
		SD	1074	370.1	815.2	1253
		Post hoc *p*-value		0.002 *	0.647	0.001 *
Fatty Acid 14:0, Myristic Acid	0.002	Mean	2467	1407	2455	1446
		SD	1344	1051	1457	1085
		Post hoc *p*-value		0.001 *	0.662	<0.0001 *
Fatty Acid 14:1 N-5, Myristoleic Acid	0.006	Mean	219.5	134.0	203.5	96.34
		SD	137.5	146.6	130.9	91.17
		Post hoc *p*-value		0.002 *	0.703	<0.0001 *
Fatty Acid 4:0, Butyric Acid	<0.0001	Mean	652.2	266.1	638.0	279.8
		SD	418.4	324.8	508.5	381.4
		Post hoc *p*-value		<0.0001 *	0.789	<0.0001 *
Fatty Acid 6:0, Caproic Acid	<0.0001	Mean	472.8	197.3	456.1	203.2
		SD	418.4	324.8	508.5	381.4
		Post hoc *p*-value		<0.0001 *	0.763	<0.0001 *
Fatty Acid 8:0, Caprylic Acid	0.001	Mean	401.6	185.1	366.4	248.4
		SD	274.7	166.7	276.8	292.5
		Post hoc *p*-value		0.001 *	0.679	<0.0001 *
Fatty acids saturated remainder	0.001	Mean	153.9	58.96	132.4	56.39
		SD	173.0	104.9	130.5	96.11
		Post hoc *p*-value		0.002 *	0.672	<0.0001 *

^a^ Individuals from the general population who reported following a diet because of an allergy were excluded from the dataset. Note. Post hoc *p*-values represent the *p*-value for the difference between the allergic populations and the general population. Note that the means and standard deviations represented in this table were not used for calculating the *p*-values; a logarithmic scale was applied for the statistical comparison. Statistically significant post hoc *p*-values are marked with an asterisk.

## Data Availability

Restrictions apply to the availability of these data. The data were originally collected by UMC Utrecht and TNO and reanalyzed using a tool developed and owned by TNO. The data are not publicly available and cannot be shared due to confidentiality and internal policy restrictions.

## Abbreviations

The following abbreviations are used in this manuscript:

ANOVA	Analysis of variance
FA	Food allergy
FDR	False discovery rate
MCFA	Medium chain fatty acids
NEVO	Nederlands Voedingsstoffenbestand
RIVM	Dutch National Institute for Public Health and the Environment
SD	Standard deviation
SCFA	Short-chain fatty acids
TNO	The Netherlands Organisation for Applied Scientific Research
UMCU	University Medical Center Utrecht
VCP	Voedsel Consumptie Peiling

## Appendix B

**Table A1:** Compounds that were not significant after FDR correction but showed a non-corrected *p*-value below 0.01. Statistically significant differences are marked with an asterisk.

Compound	*p*-Value ANOVA		No Allergy	Cow’s Milk or Hen’s Egg Allergy	Peanut or Tree Nut Allergy	Mixed Allergies
C10:1 cis	0.006	Mean	20.86	10.77	19.94	7.078
		SD	24.05	20.32	22.82	10.34
		Post hoc *p*-value		0.626	0.022	0.016
Calcium	0.006	Mean	1024	824.7	952.2	718.1
		SD	430.0	354.9	352.4	374.6
		Post hoc *p*-value		0.077	0.572	0.002
Capsaicin	0.007	Mean	75.41	216.9	104.2	137.8
		SD	94.37	157.7	159.0	83.69
		Post hoc *p*-value		0.010	0.472	0.006 *
Capsiamide|N-(13-Methyltetradecyl) Acetamide	0.007	Mean	3.959	11.39	5.471	7.231
		SD	4.955	8.279	8.349	4.394
		Post hoc *p*-value		0.010	0.472	0.006 *
Capsianoside-A	0.007	Mean	3.139	9.030	4.338	5.735
		SD	3.928	6.564	6.610	3.484
		Post hoc *p*-value		0.011	0.479	0.007 *
Capsianoside-B	0.007	Mean	0.189	0.542	0.261	0.344
		SD	0.236	0.394	0.398	0.209
		Post hoc *p*-value		0.010	0.465	0.006 *
Capsianoside-C	0.007	Mean	1.301	3.742	1.798	2.377
		SD	1.628	2.720	2.743	1.444
		Post hoc *p*-value		0.011	0.478	0.007 *
Capsianoside-D	0.007	Mean	0.556	1.600	0.769	1.016
		SD	0.696	1.163	1.173	0.617
		Post hoc *p*-value	-	0.010	0.473	0.006
Capsianoside-E	0.007	Mean	0.283	0.814	0.391	0.517
		SD	0.354	0.591	0.596	0.314
		Post hoc *p*-value	-	0.010	0.466	0.006 *
Capsianoside-F	0.007	Mean	0.094	0.271	0.130	0.172
		SD	0.118	0.197	0.199	0.105
		Post hoc *p*-value		0.010	0.475	0.006
Capsianoside-I	0.007	Mean	0.339	0.976	0.469	0.620
		SD	0.425	0.710	0.716	0.377
		Post hoc *p*-value		0.010	0.479	0.006 *
Capsianoside-II	0.007	Mean	1.706	4.908	2.358	3.117
		SD	2.135	3.568	3.598	1.893
		Post hoc *p*-value		0.010	0.470	0.006 *
Capsianoside-III	0.007	Mean	1.131	3.254	1.563	2.067
		SD	1.416	2.365	2.385	1.255
		Post hoc *p*-value		0.010	0.462	0.006 *
Capsianoside-IV	0.007	Mean	0.170	0.488	0.234	0.310
		SD	0.212	0.355	0.358	0.188
		Post hoc *p*-value		0.011	0.480	0.007 *
Capsianoside-V	0.007	Mean	0.038	0.108	0.052	0.069
		SD	0.047	0.079	0.080	0.042
		Post hoc *p*-value		0.010	0.467	0.006 *
Capsidiol	0.007	Mean	0.547	1.573	0.756	0.999
		SD	0.684	1.143	1.153	0.607
		Post hoc *p*-value		0.010	0.479	0.006 *
Cholesterol	0.007	Mean	190.2	205.3	188.6	132.0
		SD	100.8	83.08	120.9	77.03
		Post hoc *p*-value		0.437	0.670	0.005 *
Daidzein	0.007	Mean	0.659	3.009	1.146	1.541
		SD	1.640	2.986	2.711	2.371
		Post hoc *p*-value		0.001 *	0.791	0.142
Decanoic Acid-Vanillylamide	0.007	Mean	0.650	1.871	0.899	1.188
		SD	0.814	1.360	1.372	0.722
		Post hoc *p*-value		0.479	0.010	0.006 *
Di-N-Propyl-Amine	0.007	Mean	0.006	0.016	0.008	0.010
		SD	0.007	0.012	0.012	0.006
		Post hoc *p*-value		0.010	0.477	0.006 *
Dihydrocapsaicin	0.007	Mean	16.05	46.18	22.18	29.33
		SD	20.09	33.57	33.85	17.82
		Post hoc *p*-value		0.011	0.475	0.007 *
Fatty Acid 11:0, Undecanoic Acid	0.009	Mean	5.193	2.861	4.340	2.671
		SD	7.794	6.437	4.945	3.757
		Post hoc *p*-value		0.011	0.392	0.273
Fatty Acid 16:1, Trans-9-Hexadecenoic Acid	0.008	Mean	71.23	53.41	65.98	37.09
		SD	50.82	55.37	50.91	30.74
		Post hoc *p*-value		0.017	0.686	0.016
Fatty Acid 18:1, Trans-11-Octadecenoic Acid	0.006	Mean	445.6	279.1	413.6	244.0
		SD	301.9	226.9	307.3	212.2
		Post hoc *p*-value		0.019	0.654	0.007 *
Fiber—dietary total	0.009	Mean	16,120	20,832	16,256	19,589
		SD	6584	4822	6751	6376
		Post hoc *p*-value		0.008 *	0.645	0.027
Genistein	0.004	Mean	1.203	5.679	1.209	2.964
		SD	3.133	5.617	2.756	4.343
		Post hoc *p*-value		0.001 *	0.757	0.056
Homocapsaicin	0.007	Mean	0.867	2.495	1.198	1.584
		SD	1.085	1.813	1.829	0.962
		Post hoc *p*-value		0.010	0.467	0.006 *
Homodihydrocapsaicin	0.006	Mean	0.867	2.495	1.198	1.584
		SD	1.085	1.813	1.829	0.962
		Post hoc *p*-value		0.462	0.010	0.006 *
L-Dehydroascorbic Acid	0.009	Mean	354.3	1085	521.0	688.8
		SD	466.9	788.4	795.2	418.4
		Post hoc *p*-value		0.016	0.262	0.007 *
N-Propyl-Amine	0.007	Mean	0.043	0.125	0.060	0.079
		SD	0.054	0.091	0.091	0.048
		Post hoc *p*-value		0.010	0.475	0.006 *
Nonanoic Acid-Vanillylamide	0.007	Mean	0.443	1.275	0.612	0.809
		SD	0.554	0.926	0.934	0.492
		Post hoc *p*-value		0.010	0.463	0.006 *
Nordihydrocapsaicin	0.007	Mean	3.299	9.491	4.559	6.027
		SD	4.129	6.899	6.958	3.661
		Post hoc *p*-value		0.011	0.482	0.007 *
Piperidine	0.007	Mean	0.098	0.282	0.135	0.179
		SD	0.123	0.205	0.207	0.109
		Post hoc *p*-value		0.011	0.476	0.007 *
Protein animal	0.001	Mean	40,758	42,234	38,914	29,015
		SD	18,341	18,171	16,604	17,807
		Post hoc *p*-value		0.649	0.669	<0.0001 *
Pyrrolidine	0.007	Mean	0.026	0.076	0.036	0.048
		SD	0.033	0.055	0.056	0.029
		Post hoc *p*-value		0.011	0.476	0.006 *
Vitamin K2	0.001	Mean	0.023	0.077	0.021	0.055
		SD	0.019	0.247	0.012	0.211
		Post hoc *p*-value		0.583	0.629	0.001 *
